# Crocetin Mitigates Irradiation Injury in an In Vitro Model of the Pubertal Testis: Focus on Biological Effects and Molecular Mechanisms

**DOI:** 10.3390/molecules26061676

**Published:** 2021-03-17

**Authors:** Giulia Rossi, Martina Placidi, Chiara Castellini, Francesco Rea, Settimio D'Andrea, Gonzalo Luis Alonso, Giovanni Luca Gravina, Carla Tatone, Giovanna Di Emidio, Anna Maria D’Alessandro

**Affiliations:** 1 Lab of Reproductive Technologies, Department of Life, Health and Environmental Sciences, University of L’Aquila, 67100 L’Aquila, Italy; giulia.rossi1@guest.univaq.it (G.R.); martina.placidi@graduate.univaq.it (M.P.); frea@unite.it (F.R.); carla.tatone@univaq.it (C.T.); 2 Andrology Unit, Department of Life, Health and Environmental Sciences, University of L’Aquila, 67100 L’Aquila, Italy; chiara.castellini@univaq.it (C.C.); settimio.dandrea@alice.it (S.D.); 3 Química Agrícola, E.T.S.I. Agrónomos y Montes, Departamento de Ciencia y Tecnología Agroforestal y Genética, Universidad de Castilla-La Mancha, Avda. de España s/n, 02071 Albacete, Spain; gonzalo.alonso@uclm.es; 4 Laboratory of Radiobiology, Division of Radiotherapy, Department of Biotechnological and Applied Clinical Sciences, University of L’Aquila, 67100 L’Aquila, Italy; giovanniluca.gravina@univaq.it; 5 Lab of Nutritional Biochemistry, Department of Life, Health and Environmental Sciences, University of L’Aquila, 67100 L’Aquila, Italy; annamaria.dalessandro@univaq.it

**Keywords:** saffron, crocetin, pubertal testis, X-rays, radiotherapy, fertility preservation, SIRT1, HuR, oxidative stress, autophagy

## Abstract

Infertility is a potential side effect of radiotherapy and significantly affects the quality of life for adolescent cancer survivors. Very few studies have addressed in pubertal models the mechanistic events that could be targeted to provide protection from gonadotoxicity and data on potential radioprotective treatments in this peculiar period of life are elusive. In this study, we utilized an in vitro model of the mouse pubertal testis to investigate the efficacy of crocetin to counteract ionizing radiation (IR)-induced injury and potential underlying mechanisms. Present experiments provide evidence that exposure of testis fragments from pubertal mice to 2 Gy X-rays induced extensive structural and cellular damage associated with overexpression of PARP1, PCNA, SOD2 and HuR and decreased levels of SIRT1 and catalase. A twenty-four hr exposure to 50 μM crocetin pre- and post-IR significantly reduced testis injury and modulated the response to DNA damage and oxidative stress. Nevertheless, crocetin treatment did not counteract the radiation-induced changes in the expression of SIRT1, p62 and LC3II. These results increase the knowledge of mechanisms underlying radiation damage in pubertal testis and establish the use of crocetin as a fertoprotective agent against IR deleterious effects in pubertal period.

## 1. Introduction

Infertility is a potential side effect of cancer therapies and significantly affects the quality of life for survivors during their pre-reproductive and reproductive years [1,2]. Although modern radiotherapy techniques have evolved to ensure a reduction of the potential risk of infertility, radiotherapy can still result in permanent or temporary gonadal toxicity in male patients [3]. The formation of sperm by the testes through the process of spermatogenesis is highly radiosensitive. Rapidly dividing testicular germ cells are highly affected by ionizing radiation (IR) and their loss is proportional to the radiation dose. Low radiation doses can cause a profound reduction or even transient complete loss of sperms [4].

A critical period for development of reproductive organs and function and establishment of fertility potential is puberty. Spermatogenesis is known to start at very early stages of pubertal development [5,6,7] and may occur before the ability to produce an ejaculate [8]. This makes it difficult to apply fertility preservation strategies in pubertal cancer patients. Cryopreservation of ejaculated spermatozoa prior to anticancer therapy, routinely used to preserve fertility in men, represents the first line treatment in adolescents, as well [9,10]. However, for some patients it may not be possible to recover sperm prior to the onset of ablative therapies. Although semen samples can be obtained from boys from the age of 12 years onwards, the onset of sperm production (spermarche) can be very difficult to predict [11]. Since there is no reliable sensitive estimate for the presence of spermatozoa in the testes, sperm extraction from testicular tissue biopsy is not reliable [12]. Therefore, new strategies to protect male fertility against IR deleterious effects in pubertal period need to be considered. One of the possible reasons for limited progress in the field is the partial understanding of the mechanistic events that could be targeted to provide protection or repair from gonadotoxicity in this peculiar period of life. Very few studies have addressed this issue in pubertal models. Proteomics results from pubertal mice testes revealed that carbon ion radiations exert acute and chronic injury by activating early and long-term mechanisms. Most proteins that are differentially expressed in early and long-term response are involved in energy supply, endoplasmic reticulum, cell proliferation, cell cycle, antioxidant capacity and mitochondrial respiration categories. Importantly, a significant increase in ROS levels was observed clearly demonstrating that high doses of carbon ion radiations disrupt the antioxidant system in testicular tissue [13,14].

The data obtained using various models including cells, animals, and recently humans suggest that radioprotective agents working through a variety of mechanisms have the potential to decrease free radical damage produced by IR [15]. Therefore, recently, much interest has been ignited to discover antioxidants which would counteract or minimize radiation-induced testicular toxicity [16,17,18,19,20,21,22].

In this context, recent research has reported that intake of saffron, or its constituents crocin and safranal, exerts protective effects against genotoxicity associated to 1–2 Gy total body irradiation in testis of adult mice [23,24]. Crocin is the glycosylated form of crocetin, a symmetric di-carboxylic acid diterpene: about 94% of the total amount of crocetin in saffron is glycosylated to crocin and 6% is present in the free form [23,24]. Similarly to flavonoids [25], crocin shows a poor bioavailability after oral administration [26]. In the intestine, crocins are hydrolyzed to the deglycosylated trans-crocetin, which is rapidly absorbed [27,28,29]. On this basis, most of the saffron therapeutic activities have been attributed to this carotenoid, known for its elevated anti-inflammatory and free radical scavenger activity [30].

In the present study, we investigated the hypothesis that crocetin may exert a radioprotective effect by preventing IR-induced damage in testis of pubertal mice. This hypothesis is based on the knowledge of the potent anti-tumor effects of this molecule, a feature essential for a potential fertoprotective agent, and on its ability to prevent ovarian toxicity induced by cyclophosphamide, an anticancer drug with strong pro-oxidant power [31]. In this study, we utilized an in vitro culture system of prepubertal mouse testes as an experimental model of spermatogenesis to investigate the efficacy of crocetin to counteract X-ray-induced testicular injury and the mechanisms underlying IR insult and potential crocetin effects. Exposure to IR initiates a cascade of events that are based on direct DNA damage and generation of free radicals. It is well documented that oxidative stress adversely affects spermatogenesis, whereas anti-oxidative enzymes like superoxide dismutase (SOD) and catalase (CAT) protect the testicular germ cells against the oxidative stress-induced apoptosis [32]. A crucial player in sensing and modulating the testis redox status is sirtuin 1 (SIRT1), a NAD+-dependent deacetylates targeting key proteins involved in the cellular stress response [33,34]. An actor of the antioxidant and radioprotective adaptive response is HuR (human antigen R), a RNA-binding protein known to stabilize mRNAs containing AU-rich elements [35,36,37]. One of the primary repair mechanisms to resolve DNA lesions caused by IR is poly (ADP-ribose) polymerase 1 (PARP1) over-activation and intracellular NAD+ level depletion [18,38]. Pathways that mitigate the effects of DNA damage during replication include translesion synthesis and template switching under the regulation of proliferating cell nuclear antigen (PCNA) [39]. A further process that deserves investigation in this context is the autophagic pathway. Autophagy is crucial for the formation and degradation of specific structures that guarantee successful spermatogenesis, and can be induced or enhanced by various external gonadotoxic stimuli [40].

Based on the knowledge and hypotheses reported above, in this study, testes obtained from pubertal mice were exposed to 2 Gy IR in the presence or absence of crocetin and subjected to the analysis of morphological parameters associated with the integrity of germinative epithelium and to the evaluation of the molecular signaling related to IR damage response including antioxidant defenses, DNA damage response and autophagy.

## 2. Results

### 2.1. Effect of Crocetin on Tubule Diameter, Cross-Sectional Area, Seminiferous Epithelium Height and Presence of Sperm in the Lumen in Pubertal Testis Exposed to IR

**Figure 1:**
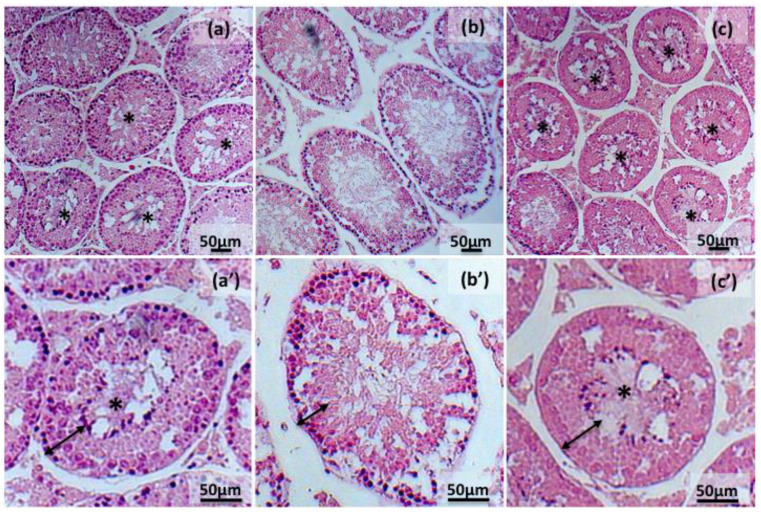
Representative images showing H&E stained testis sections from control (**a**,**a’**), irradiated (**b**,**b’**) and crocetin + irradiated group (**c**,**c’**). Histological features of irradiated testes show a significant increase in diameter and cross-sectional area of seminiferous tubules, a decrease of seminiferous epithelium height compared with the control group. Crocetin pretreatment significantly protects irradiated testes from X-rays injury. Seminiferous epithelium height is delimited by black double arrow. Mature spermatozoa located into the lumen of tubules are indicated with asterisk.

Histomorphometrical examination of testes exposed to 2 Gy X-rays showed a significant increase in diameter (195.06 ± 2.84 μm) and cross-sectional area (30.55 × 10^3^ ± 0.90 μm^2^) of seminiferous tubules associated with a decrease of seminiferous epithelium height (50.52 ± 1.21 μm) when compared with the control (CTRL) group (183.76 ± 2.39 μm; 27.04 × 10^3^ ± 0.2 μm^2^; 56.75 ± 1.04 μm, respectively, Table 1). In addition, the percentage of tubules with active spermatogenesis (29.18% ± 2.68) appeared significantly reduced in comparison to the control group (39.57% ± 2.77). Treatment with crocetin was able to counteract X-rays deleterious effects on testis when compared to the IR group. Values of mean diameter of seminiferous tubules and cross-sectional area (184.15 ± 2.73 μm; 27.51 × 10^3^ ± 0.86 μm^2^, respectively) in testes exposed to crocetin were not significantly different from the control group. Moreover, the lumen of seminiferous tubules presented a germinal epithelium with a thickness (56.14 ± 1.39 μm) similar to control. A higher percentage of tubules with active spermatogenesis (58.58% ± 3.99) was observed in comparison to control group (Table 1, Figure 1).

### 2.2. Effect of Crocetin on Protein Expression of PARP1 and PCNA in Pubertal Testis Exposed to IR

**Figure 2:**
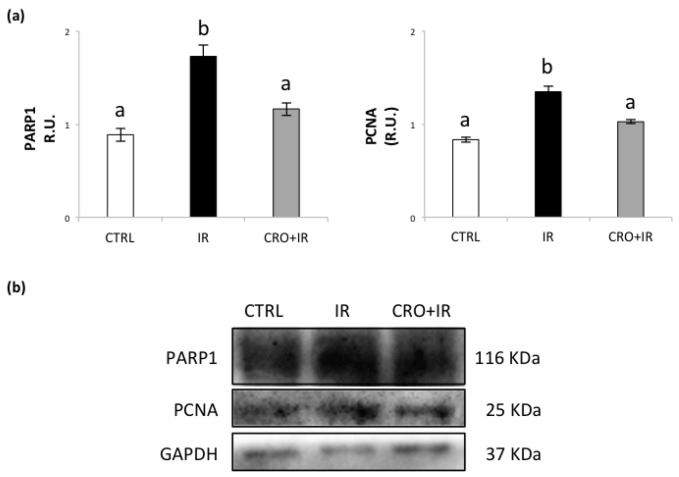
(**a**) Western blot analysis of PARP1 and PCNA protein levels. (**b**) Representative images of immunoreactive bands. Data are presented as means ± SEM of densitometric analysis of immunoreactive bands normalized to internal reference protein (GAPDH). One-way ANOVA *p* < 0.05; ^a,b^ *p* < 0.05, Holm–Sidak post hoc multiple comparison.

To investigate the IR effects on DNA, we evaluated the expression of markers involved in DNA strand breaks repair such as PCNA and PARP1. As shown in Figure 2, we detected an upregulation of PCNA and PARP1 to the control group, confirming the activation of the DNA repair response. The group treated with crocetin showed a lower amount of PCNA than the irradiated group and similar levels than control. The ability of crocetin to restore the basal levels of PARP1 and PCNA levels highlighted the establishment of a protective mechanism against radiation-induced DNA damage.

### 2.3. Effect of Crocetin on Protein Expression of SIRT1, Hur, SOD2 and CAT in Pubertal Testis Exposed to IR

**Figure 3:**
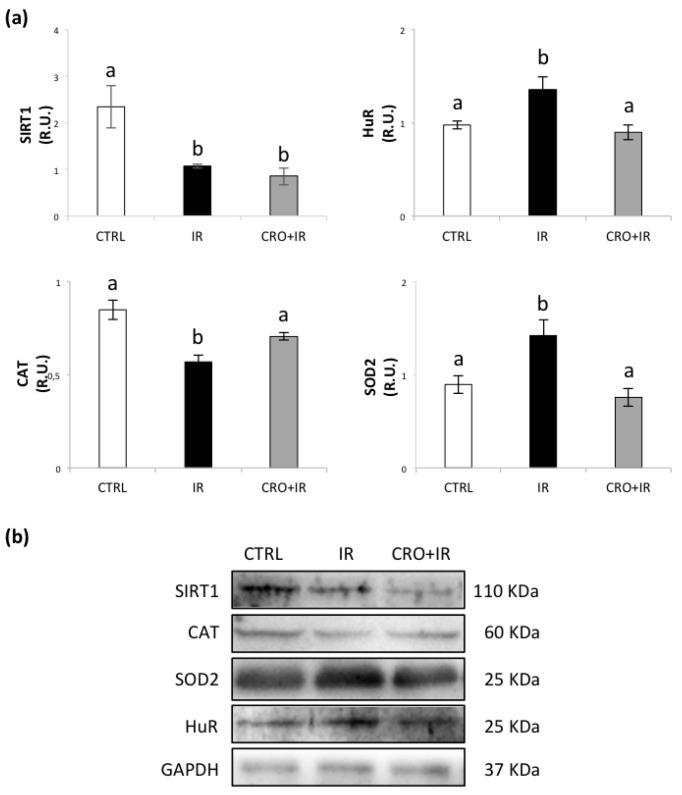
(**a**) Western blot analysis of SIRT1, HuR, CAT, and SOD2 protein levels. (**b**) Representative images of immunoreactive bands. Data are presented as means ± SEM of densitometric analysis of immunoreactive bands normalized to internal reference protein (GAPDH). One-way ANOVA *p* < 0.05; ^a,b^ *p* < 0.05, Holm–Sidak post hoc multiple comparison.

To investigate the involvement of oxidative stress in the response to IR, we analysed the levels of SIRT1, HuR, SOD2 and CAT. The group exposed to IR showed lower levels of SIRT1 and CAT and higher levels of SOD2 and HuR. The crocetin supplementation was able to restore the basal levels of SOD2, CAT and HuR but had no effects on SIRT1 expression following IR (Figure 3). The establishment of a condition of oxidative stress in IR testes was confirmed by evaluating lipid peroxidation [41,42]. As shown in Supplementary Figure S1, IR insult induced a significant increase in 4-HNE immunostaining, which was prevented by medium supplementation with crocetin.

### 2.4. Effect of Crocetin on Autophagy Markers on Pubertal Testis Exposed to IR

**Figure 4:**
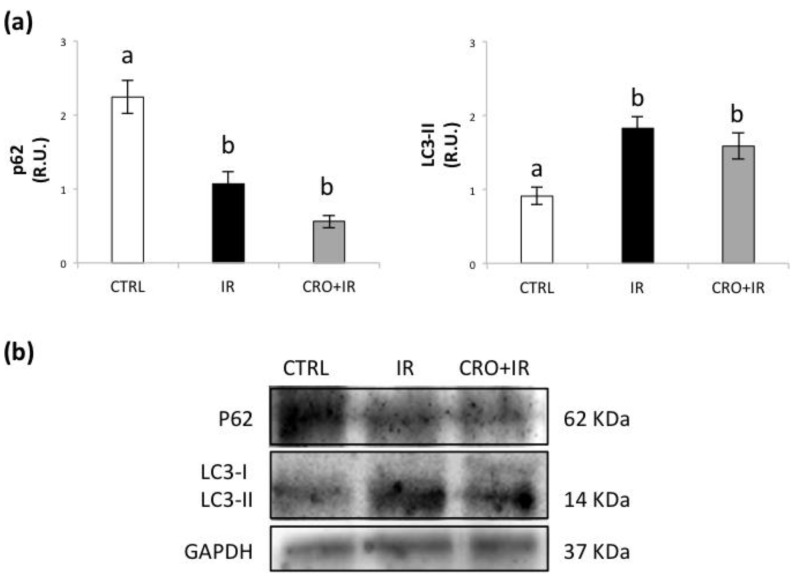
(**a**) Western blot analysis of p62 and LC3-II. (**b**) Representative images of immunoreactive bands. Data are presented as means ± SEM of densitometric analysis of immunoreactive bands normalized to internal reference protein (GAPDH). One-way ANOVA *p* < 0.05; ^a,b^ *p* < 0.05, Holm–Sidak post hoc multiple comparison.

To evaluate the role of autophagy, we analysed the proteins implicated in this process. Our data showed that IR significantly decreased the content of p62 and raised LC3-II in mice testes compared with control as an evidence of increased autophagy. Crocetin pretreatment was not capable to restore the basal levels of p62 and LC3-II and so to prevent the autophagic activation (Figure 4).

## 3. Discussion

In this study, we utilized an in vitro model of the pubertal testis as an experimental model to investigate the efficacy of crocetin to counteract IR-induced injury and potential underlying mechanisms in the pubertal male gonad. Results from our experiments provide evidence that exposure of pubertal testicular tissue to 2 Gy irradiation induced extensive structural and cellular damage associated with activation of DNA damage and antioxidant responses and induction of autophagy. A twenty-four hour exposure to 50 µM crocetin pre- and post-IR significantly reduced testis injury and modulated the response signalling pathways to cell damage.

Radioprotectors targeting oxidative damage and inflammatory reaction have been studied for decades with limited success, because of the limited effect, toxicity or risk of tumorigenesis [15]. In this context, we have hypothesized the potential of crocetin as a protective agent in relation to its antioxidant, anti-inflammatory and antitumor activities which would facilitate clinical application [43]. According to [32], the in vitro culture system selected to test IR and crocetin effects was able to sustain in vitro spermatogenesis. We observed that about 40% of tubules from pubertal testicular fragments were characterized by the presence of sperm in the lumen after 48 hr in vitro culture. In this system, the gonadotoxic effects of IR were evidenced by the appearance of enlarged seminiferous tubules in association with reduced germinal epithelium thickness and percentage of tubules with complete spermatogenesis (sperm in the lumen). By focusing on these parameters, we established that crocetin supplementation was able to counteract IR insult and sustain spermatogenesis as evidenced by an increased percentage of tubules showing sperm in the lumen. This conclusion, which represents the first evidence for crocetin radioprotective effects in the male gonad, is consistent with previous findings in adult mice receiving saffron extracts or crocin after IR [23,24] and provides evidence that the effects of saffron carotenoids intake described in these studies may be mediated by the direct action of crocetin obtained from hydrolyzation of ingested crocin [27,44].

Exposure to IR initiates a cascade of events that are based on direct DNA damage and generation of free radicals. PARP1 is involved in primary repair mechanisms to resolve DNA lesions caused by toxicants and plays an important role in safeguarding DNA integrity in spermatogenesis [45]. Gamma-irradiation-dependent increase of PARP1 activity has been recently reported in testis of adult rat [18]. For these reasons, PARP1 overexpression in irradiated pubertal testes here described is considered an evidence of the activation of a DNA damage response and/or excessive amount of ROS [46,47]. Cell response to DNA damage may involve functional association between PARP1 and PCNA [48]. Accordingly, in our experimental model, increased PARP1 protein was associated with overexpression of PCNA. The finding that PARP1 and PCNA levels did not change in irradiated testes exposed to crocetin strongly suggests that crocetin exposure is able to mitigate the IR gonadotoxic insult in this experimental model.

Radioprotective effects of crocetin observed in somatic tissues have been ascribed to elevated activities of endogenous antioxidant enzymes [49]. Here we have found that response to irradiation of pubertal testis fragments is characterized by overexpression of SOD2 and downregulation of CAT. SOD2, also known as manganese-dependent superoxide dismutase (MnSOD), is a mitochondrial protein that converts superoxide ion into oxygen and hydrogen peroxide. This, in turn, is transformed into water and oxygen by CAT. In contrast to SOD2, CAT levels were reduced in the irradiated sample, revealing an altered process of mitochondrial detoxification of the superoxide anion, resulting in a condition of oxidative stress. The observation that crocetin exposure was effective in maintaining basal protein levels of SOD2 and CAT during IR supports the hypothesis that radioprotective activity of crocetin is mediated by its ROS scavenging activity or modulation of antioxidants genes [31,48,50,51,52]. A further evidence is the observation in testes cultured in the presence of crocetin prior and after irradiation of a reduction of oxidative damage measured by levels of 4-HNE, a well-known marker of lipid peroxidation [42].

Total body exposure to IR results in reduced gene expression and activity of SIRT1, a member of the family of sirtuin, histone NAD+-dependent deacetylases, in testes of adult rats. SIRT1 plays a crucial role as a sensor of cellular energy status and oxidative stress in the male gonad [33,34]. Here we show that the pubertal testes exposed to irradiation undergo a decrease in SIRT1 protein level, a condition known to be associated to severe oxidative stress [18,33]. However, exposure to crocetin did not counteract this effect, suggesting that the radioprotective effects of crocetin are partial and not mediated by a SIRT1-dependent response. A possible explanation for decrease of SIRT1 protein level may be the dissociation of SIRT1 mRNA from the RNA-binding protein HuR, which occurs under severe oxidative stress [52,53,54]. HuR overexpression has been associated with increased resistance to damage induced by irradiation and promotion of cell survival [37]. In the testis, this protein has a fine-tuned regulation that influences post-meiotic cell formation, spermatid maturation and sperm production [35,36]. In the present study, HuR expression is increased upon IR and maintained at basal levels by exposure to crocetin, revealing HuR involvement in the adaptive response to IR in the male gonad. Therefore, the low level of SIRT1 in the crocetin group may be explained by hypothesizing additional mechanisms which are out of crocetin control.

Cellular autophagy is a cellular mechanism for selective removal of damaged cytoplasmic components. Selective autophagy has been documented to play a pro-survival role in spermatogenic cells under physiological and adverse conditions. It has been reported to minimize cell damage and promote clearance of damaged proteins and mitochondria under oxidative insult [55]. According to [56], the increase in the autophagic marker LC3-II we found in this study represents an evidence that IR-exposed testis is characterized by augmented autophagic flux. This was further confirmed by the data related to p62, which decreases when autophagy is induced [57,58]. In the testis fragments subjected to IR, there was an increase in LC3II and a decrease of p62 irrespective of the crocetin treatment. Nevertheless, our observations about the status of health of the seminiferous epithelium and the pathways related to response to DNA damage and oxidative insult may suggest that activation of autophagy in the presence of crocetin reflects a cellular effort leading to repair and maintenance of spermatogenesis. Under this condition, tissue integrity is associated with that of DNA and redox modulation. However, the lack of recovery of normal SIRT1 levels is an evidence of a sublethal damage which deserves further investigation. However, the study of the molecular pathways involved in the response to IR shows that this protective effect is partial. While protecting the germinative epithelium and modulating the response to DNA damage and oxidative stress, it does not counteract the radiation-induced decrease in SIRT1 and autophagy. These results underline the need to investigate the long-term effects of crocetin, a condition that requires in vitro experimental models capable to sustain spermatogenesis in the long term.

Overall, this study provides new insights into the short-term cellular and molecular damage caused by ionizing radiation in pubertal mouse testes and reveals crocetin ability to decrease IR injury in the pubertal mouse testis. This provides the basis for establishing new strategies to protect male fertility against IR deleterious effects in adolescent patients who are unable or unwilling to produce a semen sample.

## 4. Materials and Methods

### 4.1. Animal Care

Male pubertal CD-1 mice (28–31 days, *n* = 12, Charles River Italia s.r.l., Calco, Italy) were maintained in a temperature-controlled environment under a 12 h light/dark cycle (7.00–19.00) with free access to feed and water ad libitum. All the experiments were carried out in conformity with national and international laws and policies (EECC 86/609, OJ 358, 1 Dec 12, 1987; Italian Legislative Decree 116/92, GU n. 40, Feb 18, 1992; National Institutes of Health Guide for the Care and Use of Laboratory Animals, NIH publication No. 85-23, 1985). The project was approved by the Italian Ministry of Health and the internal committee of the University of L’Aquila. Animals were euthanized by cervical dislocation after an inhalant overdose of carbon dioxide (CO2, 10–30%), followed by cervical dislocation. All efforts were made to minimize animal suffering.

### 4.2. Crocetin Preparation

Crocetin isolation was performed by crocetin esters and purified by an internal method of the Verdù Cantò Saffron Spain Company (Novelda, Alicante, Spain) [59]. Crocetin quantification was estimated using the method based on the extinction coefficient and the related area calculated according to [60,61]. Crocetin in its free-acid form is insoluble in water and most organic solvents, except for dimethyl sulfoxide (DMSO) [62]. Crocetin was dissolved in DMSO 0.3 M and diluted in the Minimum Essential Medium-alpha (MEM-α, Euroclone, Pero, Milan, Italy) to achieve concentrations of 50 μM, prepared daily and protected from light. The final concentration of DMSO was 0.02%.

### 4.3. Mouse Testis Culture

After collection, tunica albuginea was removed and testes were cut in four pieces. Testis fragments were cultured in 12-well culture plates with polycarbonate nucleopore membrane (Whatman, Camlab Ltd., Cambridge, UK) in Minimum Essential Medium-alpha (MEMα) (Invitrogen, Thermo Fisher Scientific Inc., Merelbeke, Belgium) supplemented with 3 mg/mL bovine serum albumin (MEMα-BSA) (Sigma Aldrich, St. Louis, MO, USA) [32,63]. Cultures were conducted at 37 °C in a CO_2_ incubator with a controlled humidified atmosphere composed of 95% air and 5% CO_2_. Mouse testis fragments were randomly assigned to three experimental groups: (1) CTRL group: control testes were cultured for 24 h in MEMα-BSA, then transferred to a new plate and cultured for another 24 h in the presence of fresh culture medium; (2) IR group: testes were cultured for 24 h in MEMα-BSA, then irradiated using an X-rays linear accelerator (an Elekta 6-MV photon linear accelerator) at a dose rate of 2 Gy (200 cGy/min) at room temperature [64,65] and cultured for another 24 h in the presence of fresh culture medium; (3) CRO+IR: testes were cultured for 24 h in MEMα-BSA supplemented with 50 μM crocetin [66]. Testes were then irradiated and cultured for another 24 h in fresh culture medium containing crocetin. At the end of treatments, testes were processed to perform histological and molecular analysis.

### 4.4. Histological Staining and Morphometric Analysis

Testes were fixed in Bouin’s solution (Sigma Aldrich, St. Louis, MO, USA), embedded in paraffin blocks, cut with a Leica sliding microtome (RM 2045, Nussloch, Germany) into section of 5 μm thick, which were mounted on microscope slides. Testicular sections were dewaxed in xylene, re-hydrated in descending ethanol concentration, 100% (*v*/*v*), 90% (*v*/*v*) and 70% (*v*/*v*), stained with Haematoxylin and Eosin (H&E) according to the manufacturer’s instructions (Bio Optica, Milan, Italy) and observed by light microscopy (Zeiss Axiostar Plus, Oberkochen, Germany).

Digital images were analysed using ImageJ 1.44 p to obtain measurements of morphometric parameters as mean diameter, the cross-sectional area of round or nearly round seminiferous tubules [67,68], seminiferous epithelium height [69] and the spermatogenesis [20]. The presence of active spermatogenesis was assessed by the observation of spermatozoa in the lumen of at least 150 seminiferous tubules in each experimental group.

### 4.5. Western Blot Analysis

Pubertal testis fragments were lysed in RIPA Lysis buffer, containing 25 mM Tris-HCL pH 7.5, 150 mM NaCl, 1% Nonidet P-40, 1 mM EDTA pH 8.0, H2O, 1 mM PMSF, 1 mM sodium ortovanadate and 1% protease inhibitor cocktail, by repeated freeze/thaw cycles in liquid nitrogen. After centrifugation at 12,000 g for 20 min at 4 °C, supernatants were collected for protein analysis. The concentration of proteins was determined by a BCA protein assay kit (Pierce, Rockford, IL, USA).

Thirty micrograms of testicular proteins were resolved by 10% SDS-PAGE electrophoresis, transferred to a polyvinylidene difluoride membrane (PVDF, Sigma Aldrich, St. Louis, MO, USA) and blocked with 5% BSA (Sigma Aldrich, St. Louis, MO, USA) in Tris-buffered saline containing 0.05% Tween 20 (TBS-T) for 1 h at room temperature. After the blocking of non-specific binding site, membranes were incubated with polyclonal rabbit anti-SIRT1 antibody (Ab12193, Abcam, Cambridge, UK; 1:750), anti-SOD2 antibody (Ab86087, Abcam; 1:1000), anti-LC3A/B antibody (AB-83557, Immunological Sciences, 1:500), anti-P62/SQSTM1 antibody (AB-83779, Immunological Sciences, 1:500) or mouse monoclonal anti-HuR (SC-71290, Santa Cruz Biotechnology Inc., Dallas, TX, USA, 1:250), anti-PARP-1 (sc-74479, Santa Cruz Biotechnology, Inc., Dallas, TX, USA, 1:300), anti-PCNA (NB500-106, Novus Biologicals, Bio-Techne srl, Milan, Italy, 1:700) anti-GAPDH (TA802519, OriGene Technologies Inc., Rockville, MD, USA, 1:750), anti-CAT (200-4151, Rockland Inc., Gilbertsville, PA, USA, 1:10,000), overnight at 4 °C, followed by incubation with peroxidase (HRP)-conjugated goat anti-rabbit IgG (BA1054, Boster Biological Technology Co., Ltd., Pleasanton, CA, USA, 1:3000) or anti-mouse secondary antibody (Ab6728, Abcam, 1:2000) for 1 h and 30 min at room temperature. The specific immune complexes were detected by ECL kit (Life Technologies-Thermo Scientific, Waltham, MA, USA) using Uvitec Cambridge system (Alliance series, Cambridge, UK). Signal normalization was carried out by using GAPDH, as the loading control protein, using ImageJ 1.44 p software. Values were given as relative units (RU). All experiments were repeated three time.

### 4.6. Immunohistochemical Analysis

Paraffin-embedded sections of testes were deparaffinized and hydrated through xylenes and graded alcohol series. To increase the immunoreactivity, the sections were boiled in 10 mM citrate buffer (pH, 6.1 Bio-Optica, Milan, Italy) in a microwave at 720 W (3 cycles/3 min each). The sections were then subjected to treatment for blocking endogenous peroxidase activity (Dako). After thorough washing, sections were incubated with MOM mouse IgG blocking reagent overnight at 4 °C (Vector Laboratories) according to the manufacturer’s protocol. Then, sections were incubated with rabbit polyclonal to 4-HNE (4 Hydroxynonenal, ab46545, Abcam, 1:100) diluted in MOM diluent for 30 min, according to the Vector Laboratories instructions. 4-HNE was revealed by biotinylated anti-rabbit IgG followed by streptavidin-HRP, DAB substrate buffer and DAB (Dako kit), according to manufacturer’s instructions. Counterstaining was performed with hematoxylin (Bio-Optica, Milan, Italy). Negative controls were performed by omitting primary antibody and substituting it with MOM diluent alone. Finally, sections were dehydrated and mounted with Neomount (Merck, Darmstadt, Germany). They were observed and photographed under a Leitz Laborlux S microscope (Leica, Wetzler, Germany) equipped with an Olympus digital compact camera.

### 4.7. Statistical Analysis

Results are expressed as mean ± standard error of the mean (S.E.M.). All data were processed using the Sigma Plot 11.0 (Systat Software Inc., San Jose, CA, USA). One-way ANOVA and Holm–Sidak post hoc analyses were performed to analyse significant differences between groups. A *p* value < 0.05 was considered statistically significant.

## Figures and Tables

**Table 1:** Morphometric parameters in pubertal testis exposed to IR in the presence/absence of crocetin.

Group of Treatment	n	Mean Tubule Diameter ^1^ (μm)	n	Cross-Sectional Area ^1^ (×10^3^ μm^2^)	n	Seminiferous Epithelium ^1^ Height (μm)	n	Spermatogenesis ^1^ (%)
CTRL	120	183.76 ± 2.39 ^a^	120	27.04 ± 0.72 ^a^	57	56.75 ± 1.04 ^a^	359	39.57 ± 2.77 ^a^
IR	108	195.06 ± 2.84 ^b^	108	30.55 ± 0.90 ^b^	52	50.52 ± 1.20 ^b^	240	29.18 ± 2.68 ^b^
CRO + IR	153	184.15 ± 2.73 ^a^	153	27.51 ± 0.86 ^a^	76	56.14 ± 1.39 ^a^	324	58.58 ± 3.99 ^c^
*p* value *		*p* = 0.006		*p* = 0.01		*p* = 0.002		*p* < 0.001

^1^ Data as presented mean ± SEM. * Statistical analysis by one-way ANOVA. ^a,b,c^ Post hoc multiple comparison by Holm–Sidak. Different letters indicate *p* < 0.05.

## Data Availability

The data presented in this study are available on request from the corresponding author.

## Supplementary Materials

Supplementary Figure S1 is available online.

## Sample Availability

Samples of the compounds are available from the authors.
