# Supplementary material for: Crocetin Mitigates Irradiation Injury in an In Vitro Model of the Pubertal Testis: Focus on Biological Effects and Molecular Mechanisms
Source: Molecules. 2021 Mar 17;26(6):1676. doi: 10.3390/molecules26061676 (PMC8002482; doi:10.3390/molecules26061676)
Supplement: Supplementary file 1 [file molecules-26-01676-s001.pdf]

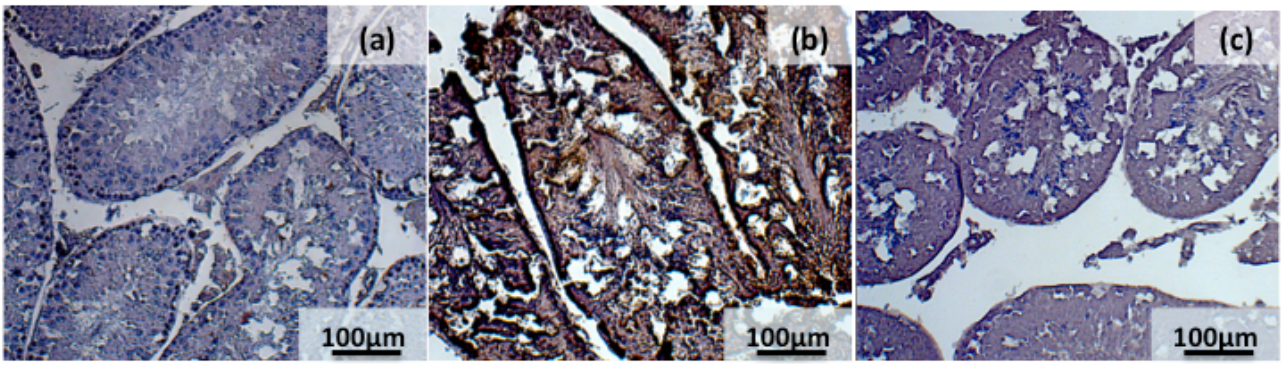

Supplementary Figure 1. Representative images showing immunolocalization of 4-HNE in control **(a)**, irradiated **(b)** and crocetin + irradiated group **(c)**.
